# Phosphatidylinositol 4,5-Bisphosphate Decreases the Concentration of Ca^2+^, Phosphatidylserine and Diacylglycerol Required for Protein Kinase C α to Reach Maximum Activity

**DOI:** 10.1371/journal.pone.0069041

**Published:** 2013-07-10

**Authors:** Antonio L. Egea-Jiménez, Ángel Pérez-Lara, Senena Corbalán-García, Juan C. Gómez-Fernández

**Affiliations:** Departamento de Bioquímica y Biología Molecular-A, Facultad de Veterinaria, Regional Campus of International Excellence "Campus Mare Nostrum", Universidad de Murcia, Murcia, Spain; University of Texas Health Science Center, United States of America

## Abstract

The C2 domain of PKCα possesses two different binding sites, one for Ca^2+^ and phosphatidylserine and a second one that binds PIP_2_ with very high affinity. The enzymatic activity of PKCα was studied by activating it with large unilamellar lipid vesicles, varying the concentration of Ca^2+^ and the contents of dioleylglycerol (DOG), phosphatidylinositol 4,5-bisphosphate (PIP_2_) and phosphadidylserine (POPS) in these model membranes. The results showed that PIP_2_ increased the *V_max_* of PKCα and, when the PIP_2_ concentration was 5 mol% of the total lipid in the membrane, the addition of 2 mol% of DOG did not increase the activity. In addition PIP_2_ decreases *K_0.5_* of Ca^2+^ more than 3-fold, that of DOG almost 5-fold and that of POPS by a half. The *K_0.5_* values of PIP_2_ amounted to only 0.11 µM in the presence of DOG and 0.39 in its absence, which is within the expected physiological range for the inner monolayer of a mammalian plasma membrane. As a consequence, PKCα may be expected to operate near its maximum capacity even in the absence of a cell signal producing diacylglycerol. Nevertheless, we have shown that the presence of DOG may also help, since the *K_0.5_* for PIP_2_ notably decreases in its presence. Taken together, these results underline the great importance of PIP_2_ in the activation of PKCα and demonstrate that in its presence, the most important cell signal for triggering the activity of this enzyme is the increase in the concentration of cytoplasmic Ca^2+^.

## Introduction

PKCα (protein kinase C α) is a classical PKC isoenzyme that is activated by second messengers, namely the increase in Ca^2+^ concentration in the cytoplasm of the cell and the appearance of diacylglycerol in the membrane, where it establishes specific interactions with phosphatidylserine and PIP_2_ [1].

**Figure 1:**
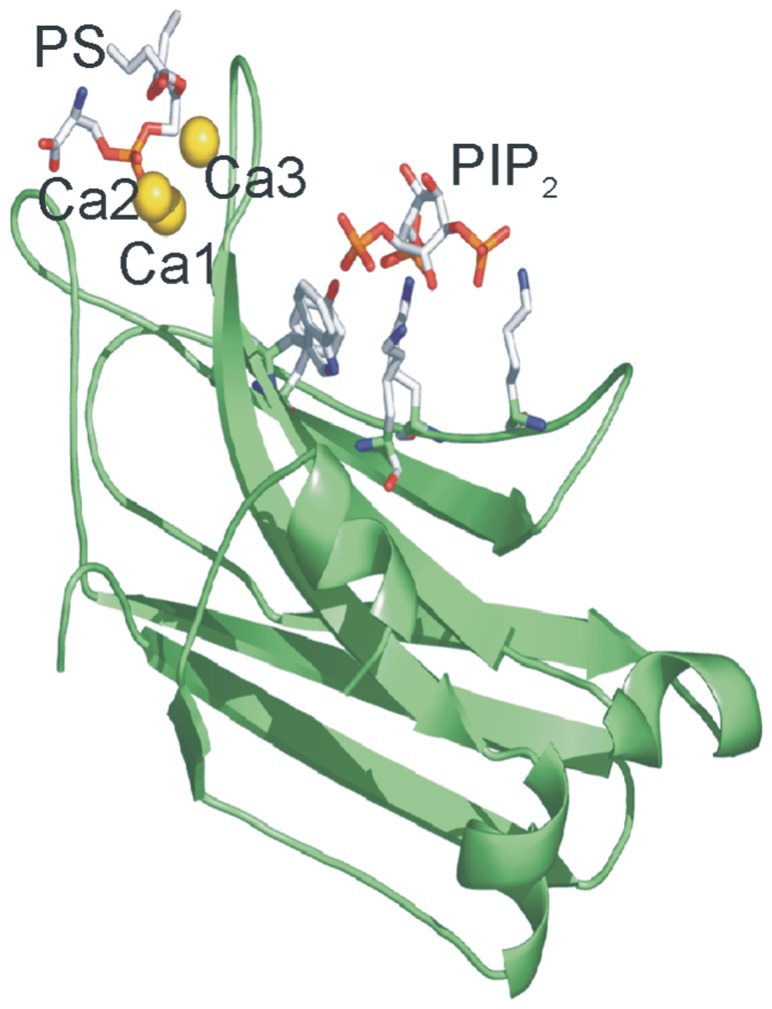
Structure of PKCα C2 domain bound to Ca^2+^-POPS-PIP_2_ in a quaternary complex. The C2 molecule is shown in green. The three calcium ions are shown in yellow spheres, one of them bridging the protein with phosphatidylserine (PS) at the tip of the domain. The PIP_2_ molecule is bound to the β3-β4 chains [8]. PDB accession number 3GPE.

The translocation of classical PKCs (cPKCs) to the plasma membrane is mediated by the C1 and C2 domains, and it has been shown that initial membrane affinity is mainly determined by C2 domain–membrane interactions, followed by C1 domain–diacylglycerol interactions [1]. One of the main sources of diacylglycerol in the plasma membrane following cell stimulation is PIP_2_ which is hydrolyzed by phospholipase C to produce diacylglycerol and inositol 1,4,5-trisphosphate, which together activate protein kinase C for sustained cellular responses [2]. However, it has been shown that PIP_2_ may also activate PKCα by direct binding to a polylysine motif located in strands β3 and β4 [3]–[7] and that can be considered a specific site for PIP_2_ [8] (see Fig. 1). Other molecules like phosphatidylserine or phosphatidic acid [9] or even retinoic acid [10] may also bind with lower affinity to this site. It has been clearly shown that PIP_2_ is important for PKCα translocation to the membrane and for prolonging this translocation. Rapid [5], [11], [12] kinetics studies on the binding of this enzyme to model membranes suggested that the interaction of PKCα with membranes occurs via two steps: a rapid weak recruitment to the membrane due to non-specific interactions with (primarily) anionic lipids and the formation of a high affinity complex due to stereospecific interactions of each PKCα domain with its specific ligands [12].

PKCα enzyme is a paradigmatic example for bearing a C2 domain which may simultaneously bind three different activators, in this case Ca^2+^, phosphatidylserine and PIP_2_. Fig. 1 shows this C2 domain in which Ca^2+^ binds to its site, acting as a bridge for phosphatidylserine, although this phospholipid also directly interacts with several protein residues [13], [14]. In another site located in a β-groove, PIP_2_ binds with great affinity.

Previous work has shown that PKCα exhibits high cooperativity in its activity by phosphatidylserine [15], [16] and that the two second messengers of the kinase, diacylglycerol and Ca^2+^, markedly increase the affinity of the kinase for phosphatidylserine [17]. In this paper, we use highly purified full-length PKCα to perform a kinetic study of the activation of PKCα by model membranes, in which the concentrations of POPS, DOG, PIP_2_ and Ca^2+^ are varied. Our results indicate that PIP_2_ enhances PKCα activity and decreases the required concentrations of the other activators, to reach maximum activities.

## Materials and Methods

### Materials

1-Palmitoyl-2-oleoyl-*sn*-glycero-3-phosphoserine (POPS), 1-palmitoyl-2-oleoyl-*sn*- glycero-3-phosphocholine (POPC), L-α-phosphatidylinositol 4,5-bisphosphate (PIP_2_) and 1,2-*sn*-dioleoylglycerol (DOG) were purchased from Avanti Polar Lipids (Alabaster, ALA, U.S.A.). To discard any 1,2-diacyl-*sn*-glycerol contamination of the PIP_2_ source used, L-α-phosphatidylinositol 4,5-bisphosphate (PIP_2_) (lot BPIP2 (4,5)-54) was analyzed by Avant Polar Lipids and their release testing included Gravimetric analysis, Phosphorus analysis, Proton and Phosphorus NMR, Mass Spectrometry, Thin Layer Chromatography (TLC), HPLC and Calcium by ICP/MS without detecting any impurity. The HPLC assay indicated 100% PIP_2_. In addition, once dissolved it was also analysed in our laboratory by using TLC and no impurity was detected either. All other chemicals were of high purity and supplied by Sigma Chemical Co. (Madrid, Spain).

### Expression and Purification of Protein Kinase Cα

The full length cDNA for rat PKCα was kindly provided by Profs. Ono and Nishizuka (Kobe, Japan). PKCα was cloned into the plasmid pFastBac HT (Invitrogen, Madrid, Spain). A 0.5 litre scale culture of Sf9 insect cells (*Spodoptera frugiperda*) at 2.1×106 cells/ml was infected with the recombinant baculovirus.Cells were harvested 60 h postinfection (cell viability 80%), pelleted at 4500 rpm for 20 min, and resuspended in buffer containing 25 mM Tris-HCl pH 7.5, 100 µM EGTA, 50 mM NaF, 100 µM NaVO_3_, 1% Triton X-100, 10% glycerol, 150 mM NaCl, 1 mM PMSF, 10 µg/ml leupeptin and 10 mM benzamidine. The pellet was disrupted by sonication (6×10 s) and the resulting lysate was centrifuged at 15000 rpm for 20 min. The supernatant was applied to a 1 ml His-Gravi TrapTM® column (GE Healthcare, Barcelona, Spain) and equilibrated with 25 mM Tris-HCl pH 7.5, 150 mM NaCl and 20 mM imidazole buffer. The bound proteins were eluted by an imidazole gradient (20–500 mM). Fractions containing protein kinase Cα from a His-Gravi TrapTM® column were pooled, concentrated by ultrafiltration to a 2 mL volume and adjusted by the addition of 5 M NaCl to give a NaCl concentration of 1 M.

This fraction was then processed by hydrophobic exchange chromatography, directly applying it to a SOURCE 15PHE 4.6/100 PE ® colum®M Tris-HCl pH 7.5, 1 mM DTT and 10% glycerol. After the unbound proteins had passed through the column, PKC was eluted with a gradient of 0.8–0 M NaCl. Highly pure PKCα was obtained, as determined by SDS-PAGE (12.5%). The protein was aliquoted and stored at −80°C in the presence of 10% (w/v) glycerol and 0.05% (v/v) Triton X-100.

### Preparation of Phospholipid Vesicles

Lipid vesicles were generated by mixing chloroform solutions of 1-palmitoyl-2-oleoyl-*sn*-glycero-3-phosphocholine (POPC), 1-palmitoyl-2-oleoyl-*sn*-glycero-3-phosphoserine (POPS) and L-α-phosphatidylinositol-4,5-bisphosphate (PIP_2_) in the desired proportions. Lipids were dried from the organic solvent under a stream of oxygen-free nitrogen, and then the last traces of organic solvent were removed under vacuum for at least 4 h. Dried phospholipids were resuspended in the corresponding buffers by vigorous vortexing and then large unilamellar phosholipid vesicles of about 100 nm diameter were prepared by extruding (11 times) rehydrated phospholipid suspensions through two stacked 0.1 nm polycarbonate membranes (Millipore Inc., Bedford, MA, USA).

### Enzymatic Activity Assay

Enzymatic activity was assayed using a technique described previously [17], in which the incorporation of radioactive phosphate [γ-^32^P] to kinase substrate (histone III-S) was measured. Lipids in organic solutions were mixed in the desired proportions and dried under a stream of N_2_, removing the last traces of organic solvent by keeping the samples under vacuum for at least four hours. Large unilamellar vesicles (LUVs) were prepared using the extrusion technique, as explained above. These lipids were resuspended immediately before use in a buffer composed of 20 mM Tris-HCl (pH 7.5), 0.5 mg/ml of histone III-S, 40 µM ATP [γ-^32^P] (3000,000 cpm/nmol), 5 mM MgCl_2_ 1 mM EGTA and enough CaCl_2_ to give a free Ca^2+^ concentration of 200 µM, except in the assays in which this concentration was varied. The necessary concentrations of CaCl_2_ added in each case were calculated by using the procedure described in [18]. The final concentration of lipids in the reaction mixture was 625 µM. The reaction was started by the addition of 5 µl of PKCα (0.004 µg/ml). After 30 min at 25°C, the reaction was stopped with 1 ml of ice-cold 25% (w/v) trichloroacetic acid (TCA) and 1 ml of ice-cold 0.05% (w/v) bovine serum albumin. After precipitation on ice for 30 min, the protein precipitate was collected on a 2.5 cm glass filter (Sartorius, Göttingen, Germany) and washed with 10 ml of ice-cold 10% trichloroacetic acid. The amount of ^32^Pi incorporated in histone was measured by liquid scintillation counting. The linearity of the assay was confirmed from the time-course of histone phosphorylation over a 30 min period. Additional control experiments were run in the absence of calcium to measure basal kinase activity only adding EGTA without any CaCl_2_ with a reaction time of 30 minutes.

### Data Analysis

The dependence of PKCα activity on the contents of the different activators in the model membranes was analyzed by a non-linear least squares fit to a modified Hill equation:
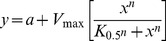
where *y* is the measured activity of PKCα, *a* is the activity in the absence of lipid or Ca^2+^ (background), *V_max_* is the lipid-stimulated activity, *x* is the concentration of the activator, *K_0.5_* is the concentration of activator resulting in half maximal activity and *n* is the Hill coefficient. Standard errors for *n*, *V_max_* and *K_0.5_*, taken for three independent experiments, are reported.

## Results

The important contribution of PIP_2_ to PKCα enzymatic activity was clearly observed when it was studied as a function of Ca^2+^ concentration. A POPC/POPS molar ratio of about 4 was used in these assays since the concentration of POPS in the inner monolayer of eukaryotic plasma membranes, such as in erythrocyte or platelet cells, is roughly this [19]–[21]. The physiological concentration of PIP_2_ has been described to be around 1 mol% of the total lipid of plasma membranes [22], [23] and it is likely to be concentrated in the inner monolayer at 2 mol%, which increase locally if it forms clusters or patches [24]. As regards diacylglycerol, the physiological levels of this lipid in biomembranes were reviewed in [25]. For example, quantitative measurements of diacylglycerols present in stimulated cells have shown that they may reach 1.45 mol% of the total lipid concentration [26] or about 2 mol% [27]. So the concentrations of diacylglycerol used in this work can be considered physiological and well within the range of diacylglycerol concentrations used in standard procedures for PKC activation assays, which use values similar to those used here [28] or even as high as 11.5 mol% with respect to total lipid [29] or as 19 mol% [30] or 25 mol% [31]. In enzymatic studies where the effect of lipid concentrations were studied, 200 µM Ca^2+^ was used in order to ensure that this cation was not a limiting factor.

**Figure 2:**
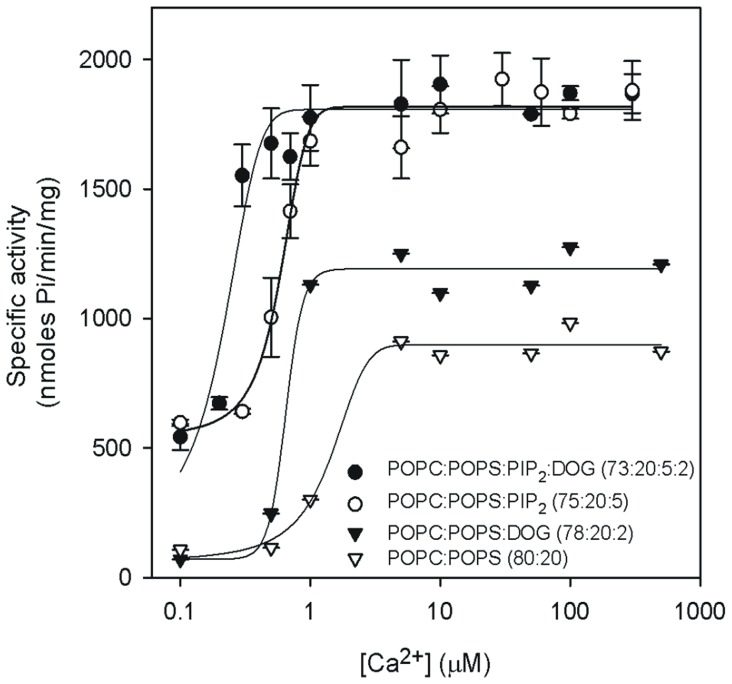
The dependence of PKCα activity on Ca^2+^ concentration. The molar ratios of the lipid components of the vesicles used to activate the enzyme are shown. Ca^2+^ concentration was 200 µmol. SD calculated from 3 independent experiments.

When the dependence of PKCα activity on Ca^2+^ concentration was studied (Fig. 2) the sigmoidal curves obtained in all cases, pointed to cooperativity. In the presence of POPC/POPS (80∶20 molar ratio) alone, increasing concentrations of Ca^2+^ led to a cooperative increase in activity, with a *K_0.5_* of 1.30 µM in Ca^2+^ (see Table 1), rising from 107.6 nmol Pi/min/mg at 0.1 µM in Ca^2+^ to a *V_max_* of 898.4 nmol Pi/min/mg and a Hill coefficient of 2.28. If DOG was added to the membrane to give a composition of POPC/POPS/DOG (78∶20∶2 molar ratio) the cooperative behavior was again present, but now the *K*_0.5_ was 0.84 µM in Ca^2+^, with the activity raising from 70.5 nmol Pi/min/mg at 0.1 µM Ca^2+^ to a *V_max_* of 1192.7 nmol Pi/min/mg and a Hill coefficient of 2.42. It is clear that in the presence of DOG, the *K_0.5_* for Ca^2+^ decreases, and there is an increase in *V_max_*.

**Table 1:** Dependence of PKCα activation on Ca^2+^.

Lipid mixture	*K_0.5_*	*V_max_* (nmol Pi/min/mg)	*n*
POPC:POPS (80∶20)	1.30±0.08	898.2±27.8	2.28±0.89
POPC:POPS:DOG (78∶20∶2)	0.84±0.27	1192.7±96.3	2.42±0.69
POPC:POPS:PIP_2_ (75∶20∶5)	0.58±0.09	1790.7±64.4	4.10±0.92
POPC:POPS:PIP_2_:DOG (73∶20∶5∶2)	0.26±0.01	1804.9±104.4	8.61±0.27

*K_0.5_*, *n* and *V_max_* were obtained by nonlinear least square fit of the data in the equation described in the Methods Section.

When PIP_2_ was added to the membrane to give a composition of POPC/POPS/PIP_2_ (75∶20∶5 molar ratio), *K*_0.5_ was now 0.59 µM of Ca^2+^ (Table 1), the activity was 597.4 nmol Pi/min/mg at 0.1 µM of Ca^2+^ and *V_max_* was 1790.7 nmol Pi/min/mg at 10 µM of Ca^2+^, the higher cooperativity giving a Hill coefficient of 4.10. Even higher cooperativity (Hill coefficient of 8.61) was observed for a membrane which incorporated also DOG, namely PC/PS/PIP_2_/DOG (73∶20∶5∶2 molar ratio), although the activity levels did not change with respect to the membrane without DOG, being now 512.9 nmol Pi/min/mg at 0.1 µM of Ca^2+^ while *V_max_* was 1804.9 nmol Pi/min/mg at 10 µM of Ca^2+^. It is interesting that at very low concentration of Ca^2+^, e.g. 0.1 µM, the activity in the presence of PIP_2_ was higher than in the absence of this phosphoinositide, both in the presence and in the absence of DOG. However, the addition of 2% DOG did not increase the activity levels when 5 mol% PIP_2_ was present.

**Figure 3:**
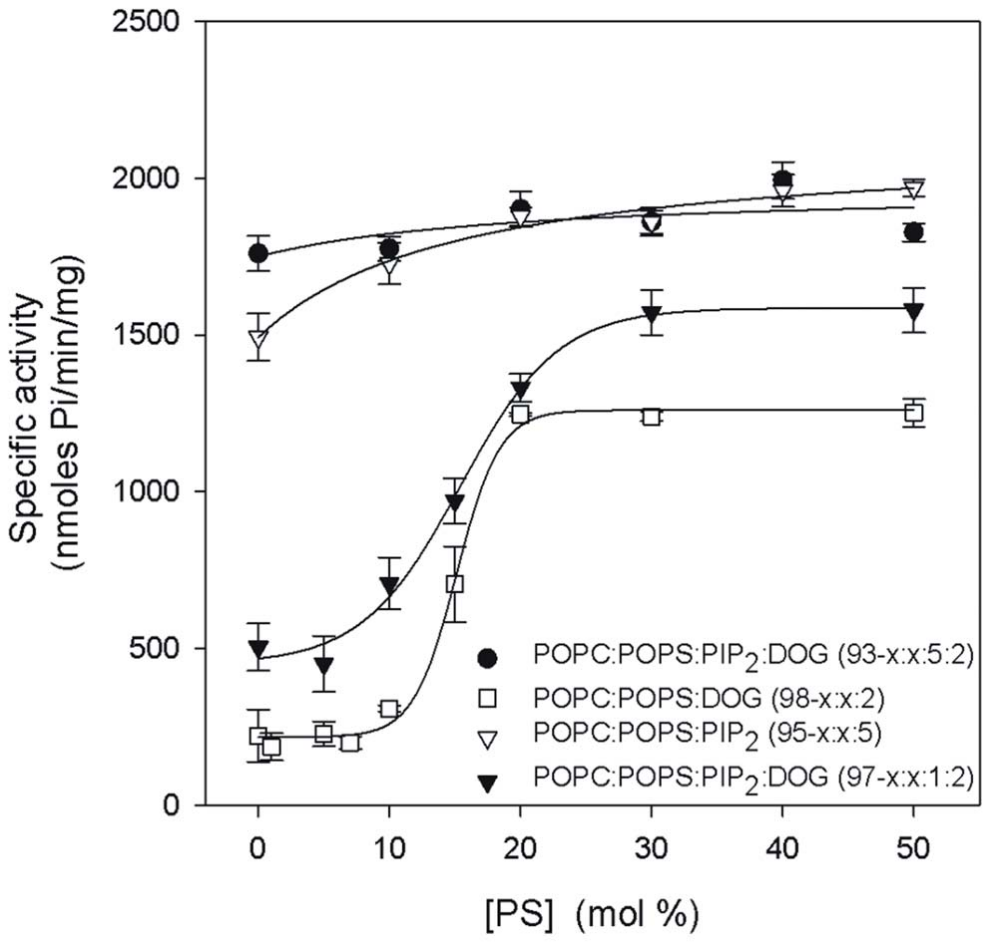
The dependence of PKCα activity on the POPS molar percentage in the vesicles. The molar ratios of the lipid components of the vesicles used to activate the enzyme are shown. Ca^2+^ concentration was 200 µmol. SD calculated from 3 independent experiments.

The effect of increasing POPS concentration was also studied (Fig. 3). When PIP_2_ was absent in a POPC/POPS/DOG mixture (98-x:x:2 molar ratio), the effect observed was of positive cooperativity (very high Hill coefficient of 13.18), with a *K_0.5_* = 15.12 mol% of POPS (Table 2), which is similar to the effect observed previously for membrane activation of this enzyme [14]. *V_max_* was 1260.2 nmol Pi/min/mg.

**Table 2:** Dependence of PKCα activation on POPS.

Lipid mixture	*K_0.5_*	*V_max_* (nmol Pi/min/mg)	*n*
POPC:POPS:DOG (98-x:x:2)	15.12±0.24	1260.2±48.4	13.18±1.9
POPC:POPS:PIP2:DOG (97-x:x:1∶2)	15.40±0.71	1586.2±71.1	3.71±0.77
POPC:POPS:PIP_2_ (95-x:x:5)	13.94±1.45	2084.0±73.6	1.10±0.39
POPC:POPS:PIP_2_:DOG (93-x:x:5∶2)	8.20±0.84	1895.8±101.3	1.50±0.56

*K_0.5_*, *n* and *V_max_* were obtained by nonlinear least square fit of the data in the equation described in the Methods Section.

If PIP_2_ was also present, at just 1 mol%, in a mixture containing POPC/POPS/PIP_2_/DOG (97-x:x:1∶2 molar ratio),positive cooperativity was still observed, with a Hill coefficient of 3.71, an initial activity of 490 nmol Pi/min/mg in the absence of POPS and a *V_max_* of 1586.2 nmol Pi/min/mg. *K_0.5_* was 15.4 mol% of POPS.

If PIP_2_ was present in the lipid mixture, but with no DOG, POPC/POPS/PIP_2_ (95-x:x:5), the initial activity, even in the absence of POPS, was already high, with a value of 1492 nmol Pi/min/mg. *V_max_* reached a value of 2084.1 nmol Pi/min/mg and *K_0.5_* was 13.94 mol% of POPS. Thus the addition of PIP_2_ decreased *K_0.5_* even if DOG was not present, and the activity was almost saturated and no apparent cooperativity was observed (*n* = 1.10).

Fig. 3 also shows that when PIP_2_ was increased to 5 mol%, to give a lipid mixture of POPC/POPS/PIP_2_/DOG (93-x:x:5∶2 molar ratio), a very small increase in activity was already observed when POPS was increased since nearly maximum activity was observed in the absence of POPS (1760 nmol Pi/min/mg) and *V_max_* was 1895.8 nmol Pi/min/mg, with *K_0.5_* of 8.20 mol% POPS and a Hill coefficient of 1.50.

**Figure 4:**
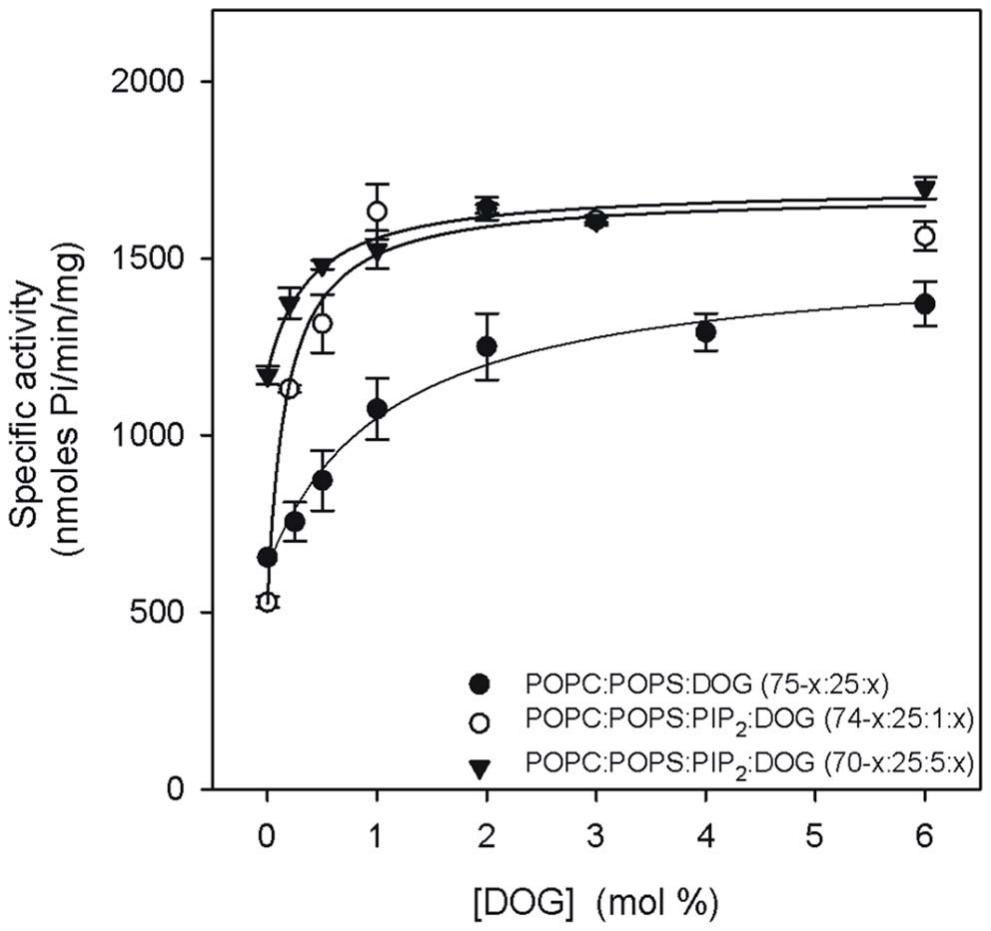
The dependence of PKCα activity on the DOG molar percentage in the lipid vesicles. The molar ratios of the lipid components of the vesicles used to activate the enzyme are shown. Ca^2+^ concentration was 200 µmol. SD calculated from 3 independent experiments.

Fig. 4 shows the activity studied as function of DOG concentration. When the membrane was composed of POPC/POPS/DOG (75-x:25:x), *K_0.5_* was 0.82 mol% of DOG (Table 3). The activity was 666 nmol Pi/min/mg at 0 mol% of DOG and rose to give a *V_max_* of 1307.9 nmol Pi/min/mg and a Hill coefficient of 1.59, indicating low positive cooperativity. When PIP_2_ was incorporated into this assay at just 1 mol% in a POPC/POPS/PIP2/DOG (74-x:25∶1:x molar ratio) mixture, *K_0.5_* was 0.38 mol% of DOG, which was notably lower than the 1.10 mol% observed in the absence of PIP_2_, while the Hill coefficient showed little change (1.39). When the PIP_2_ concentration was raised to 5 mol%, to give a POPC/POPS/PIP_2_/DOG mixture (70-x:25∶5:x molar ratio), *K_0.5_* decreased to 0.17 mol% DOG, although *V_max_* maintained a similar value of 1701.9 nmol Pi/min/mg and the Hill coefficient (0.47) indicated an apparent negative cooperativity.

**Table 3:** Dependence of PKCα activation on DOG.

Lipid mixture	*K_0.5_*	*V_max_* (nmol Pi/min/mg)	*n*
POPC:POPS:DOG (75-x:25:x)	0.82±0.08	1307.9±52.7	1.59±0.22
POPC:POPS:PIP2:DOG (74-x:25∶1:x)	0.38±0.10	1681.7±85.9	1.39±0.01
POPC:POPS:PIP2:DOG (70-x:25∶5:x)	0.17±0.05	1701.9±69.2	0.47±0.15

*K_0.5_*, *n* and *V_max_* were obtained by nonlinear least square fit of the data in the equation described in the Methods Section.

**Figure 5:**
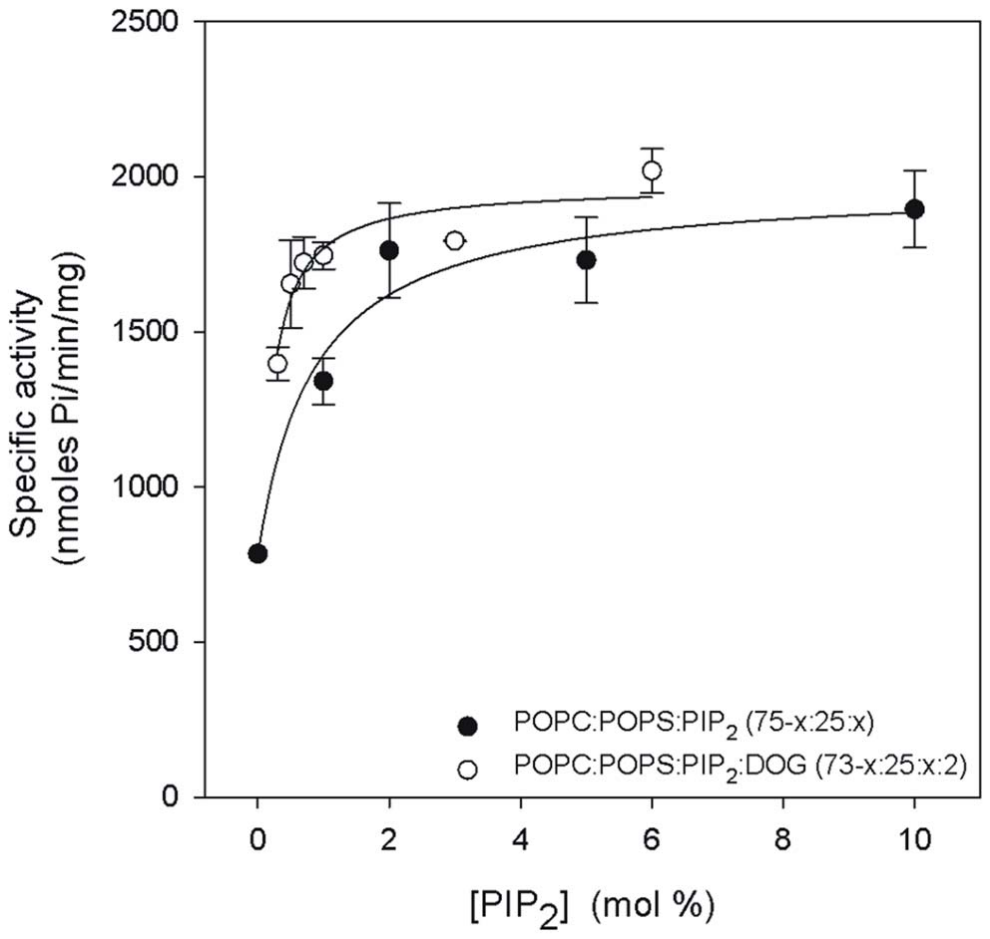
The dependence of PKCα activity on the PIP_2_ molar percentage in the lipid vesicles. The molar ratios of the lipid components of the vesicles used to activate the enzyme are shown. Ca^2+^ concentration was 200 µmol. SD calculated from 3 independent experiments.

In another set of experiments, the concentration of PIP_2_ was varied in the presence and in the absence of DOG, keeping the Ca^2+^ concentration constant at 200 µM. Fig. 5 depicts the results obtained when the molar percentage in the membrane of PIP_2_ was increased in the absence of DOG, POPC/POPS/DOG (75-x:25:x). As can be seen, the *K_0.5_* was 0.39 (Table 4) and the *V_max_* 1816.2 nmol Pi/min/mg, with low positive cooperativity (*n* = 1.60). In the presence of 2 mol% with a POPC/POPS/PIP_2_/DOG membrane (73-x:25:x:2, molar ratio) *K_0.5_* decreased to 0.11 mol% PIP_2_. This is interesting since it clearly demonstrates that very low concentrations (well below physiological concentrations) are sufficient to significantly enhance the activity of PKCα. *V_max_* was 1857.6 nmol Pi/min/mg in this case, which is not significantly differrent with respect to the mixture without DOG, but the Hill coefficient was now 0.78, indicating apparent negative cooperativity during PIP_2_ activation.

**Table 4:** Dependence of PKCα activation on PIP_2_.

Lipid mixture	*K_0.5_*	*V_max_*(nmol Pi/min/mg)	*n*
POPC:POPS:PIP_2_ (75-x:25:x)	0.39±0.61	1816.2±143.5	1.60±0.03
POPC:POPS:PIP_2_:DOG (73-x:25:x:2)	0.11±0.02	1857.6±73.9	0.78±0.15

*K_0.5_*, *n* and *V_max_* were obtained by nonlinear least square fit of the data in the equation described in the Methods Section.

## Discussion

This study was designed to show the importance of PIP_2_ for the catalytic activity of PKCα. Previous studies have shown that PIP_2_ binds to a site located in the β3- and β4-sheets of the C2 domain, which is a conserved site found also in other C2 domains (Fig. 1) [8]. It was also shown that the presence of PIP_2_ considerably increased the kinase activity of PKCα [3]. More recently the binding of PKCα to model membranes was studied by monitoring rapid kinetics [12]. It was observed that, in the absence of PIP_2_, the protein rapid rate of binding was especially dependent on the POPS concentration and a high affinity complex evolved more slowly, which implies specific interactions with POPS and DOG. Both association and dissociation constants were decreased by the presence of PIP_2_, but the association equilibrium constant was increased, i.e. the species of PKCα bound to the membrane was favored. A model was proposed in which PKCα binds to the membranes via a two-step mechanism consisting of a rapid initial recruitment of PKCα to the membrane driven by interactions with POPS and/or PIP_2_, although interactions with DOG are also involved. PKCα carries out a 2-D search on the lipid bilayer to establish specific interactions with its specific ligands. In this way the longer time of residence of the enzyme in the bound state induced by PIP_2_ could explain its activating effect.

In this work we have assayed in detail the enzymatic activity of PKCα changing the concentrations of the different activators in order to obtain a comprehensive picture of the way in which PIP_2_ may affect the catalytic action of this enzyme.

With respect to the dependence on Ca^2+^, it was observed that, as it has long been known [32], the addition of DOG to POPC/POPS increased to activity, the *V_max_* going from 898.2 to 1192.7 nmol Pi/min.mg (Table 1), and decreased *K_0.5_* [33], but the Hill coefficient was not significantly altered, showing positive cooperativity. Therefore, the binding of DOG to the C1 domain will influence Ca^2+^ binding to the C2 domain, facilitating the binding of the C2 domain to the membrane at lower Ca^2+^ concentrations, an increase in activity that can only be due to its capacity to retain the C1 domain bound to the membrane, impeding return to the inactive structure of PKC.

The addition of PIP_2_ in the absence of DOG increased *V_max_* with respect to a membrane containing POPC/POPS/DOG (1790.7 compared to 1192.7 nmol Pi/min.mg) and the further addition of DOG led to the maximum activation (*V_max_* reaching 1804.9 nmol Pi/min.mg). However, the effect of DOG in this case was not great since the activity was already close to the maximum in the absence of DOG. Note that when PIP_2_ is introduced and POPC/POPS/DOG and POPC/POPS/DOG/PIP_2_ are compared, the *K_0.5_* for Ca^2+^ is reduced in the presence of PIP_2_ more than three-fold (from 0.84 to 0.26 µM) and the Hill coefficient increases from 2.42 to 8.61. Since it is known that the presence of PIP_2_ collaborates in the anchoring of the C2 domain to the membrane [3], [8], [12], [34] and that Ca^2+^ is necessary to allow the binding of the protein to the membrane, it is clear that there is an interplay between these activators. It seems that the presence of PIP_2_ lowers the amount of Ca^2+^ required for binding and activity and, at the same time, increases the cooperativity for the binding of Ca^2+^. Indeed, it has been shown that PIP_2_ markedly reduces the concentration of Ca^2+^ required for the binding of isolated C2 domain [4], [34]. It is known that up to 3 Ca^2+^ ions may bind at the calcium binding site of the C2 domain of PKCα [9], [35]. A sequential model for classical PKC membrane binding and activation has been proposed [3], [5], [36], whereby the increase in intracellular Ca^2+^ produces the binding of Ca1 and Ca2 when the protein is still in the cytosol, leading to the membrane being targeted by the enzyme through the C2 domain. Ca1 is responsible for bridging the protein to the phospholipid molecules (Fig. 1), which are also recognized with the help of Asn189 and Arg216, whereas Ca2 is responsible for keeping Ca1 in its proper location and for inducing a conformational change in PKC, which partially penetrates and docks in the phospholipid bilayer by means of CBR3 (Arg249 and Thr251). Once recruited to the membrane, a third Ca^2+^ binds, stabilizing the C2 domain-membrane complex. This enables PKC to reside in the membrane for a longer time, allowing the C1 domain to find the diacylglycerol generated upon receptor stimulation [14], [33], [37]–[43].

In the case of PKCα dependence on POPS, *V_max_*, was clearly increased by the addition of 1 mol% of PIP_2_, and the addition of 5 mol% in the absence of DOG led to very high activities, which did not increase even when DOG was added. This is an interesting result and confirms the great activation capacity of PIP_2_, and shows that fixing the C2 domain of PKCα to the membrane through the calcium binding site and the PIP_2_ site decreases the importance of the C1 domain respect to activity. However, the addition of DOG to the membrane containing 5 mol% PIP_2_ reduced *K_0.5_* from 13.94 to 8.20 µM, showing that binding of the C1 domain may also play a role.

It has been described that POPS binds cooperatively to PKC, with a stoichiometry of 4 [44], ≥12 [15] or approximately 8 [45] lipid molecules per molecule of protein. A number of authors have observed apparent cooperativity for the activation in mixed micelles with Triton X-100, leading to high Hill coefficients (higher than 8 [46]–[48] or about 5 [49]) but when the activation was carried out with lipid vesicles, the Hill coefficients were about 2.6 [50] or close to 1 [49]. In our case, a high degree of positive cooperativity was observed in the absence of PIP_2_, with a Hill coefficient of 13.18. The use of different types of vesicles in the studies mentioned above may be the reason for the disparity of the results. However, the addition of just 1 mol% of PIP_2_ reduced *n* to 3.71, and at 5 mol% PIP_2_ no cooperativity was evident. Just one POPS molecule is known to bind to the C2 domain of PKCα [8], although more POPS molecules may bind to the C1 domain [51]. It is interesting in this respect that Hill coefficients close to 1 were observed for the binding of the isolated C2 domain to phospholipid vesicles (A. Torrecillas, Ph.D. Thesis, University of Murcia, 2003).

However, it is nowadays recognized that a number of mechanisms may lead to kinetic cooperativity in the absence of true cooperative interactions, and kinetic models have been suggested to explain the apparent cooperativity observed for the binding of lipid to PKC, for example, proposing ligand trapping [52] or effects specific to the interaction with multiple membrane associated ligands [45] have been suggested, the last authors observing that the apparent cooperativity may be abolished in conditions where membrane binding involves a non-phosphatidylserine mechanism, as in the presence of activators such as phorbol esters. This explains why PIP_2_ reduces the apparent cooperativity so drastically. Therefore, Hill indexes obtained for the binding of proteins, such as PKC to lipids in vesicles or in micelles, may be informative as regards threshold-binding and how this type of binding may be regulated by different ligands.

Diacylglycerol also plays a role, especially in the absence of PIP_2_, but in the presence of the phosphoinositide its role is reduced. The reason for that may be related to the tighter anchoring of the enzyme as seen by stopped flow experiments using the full-length enzyme [12] and the different orientation of the C2 domain with respect to the membrane, as seen by studying the membrane docking of this domain [53]. These effects occasioned by the interaction with PIP_2_ may prolong the activated state. The interplay between DOG and PIP_2_ was also evident when DOG was changed. Even at 1 mol% of PIP_2_, *K_0.5_* decreased from 1.10 mol% of DOG in its absence to 0.38 in its presence. If PIP_2_ was 5 mol%, then *K_0.5_* further decreased to 0.17 mol% of DOG, a substantial decrease compared with the total absence of the phosphoinositide. This illustrates that in the presence of PIP_2_ the enzyme is tightly bound to the membrane and small concentrations of DOG are sufficient to maintain the activity. It is interesting that small apparent positive cooperativity was detected in the absence of PIP_2_ (*n* = 1.59), which was reduced following the addition of 1 mol% PIP_2_. This apparent cooperativity in the binding of DOG may not necessarily reflect that the two C1 subdomains bind to DOG when its concentration is sufficiently high, but may be explained by the apparent cooperativity effect described above for POPS, while PIP_2_ will reduce the apparent cooperativity due to its increasing of the membrane affinity of the protein. It is remarkable, that at 5 mol% of PIP_2_ the Hill coefficient (0.47) indicated apparent negative cooperativity for diacylglycerol, which might be explained by a change in membrane structure at relatively high DOG concentrations [25].

It is interesting that very low *K_0.5_* values were observed for PIP_2_ even in the absence of DOG, the value (0.39 µM) being within the physiological range of concentrations. In the presence of DOG a very reduced *K_0.5_* value of 0.11 µM was observed for PIP_2_, although *V_max_* increased very slightly as a result of the addition of DOG, confirming that in the presence of PIP_2_ diacylglycerol is playing a relatively secondary role in the activation of PKCα. Low *K_D_* values have been reported for the binding of PIP_2_ to the isolated C2 domain of PKCα [4], [54] with about 1.9 µM for POPC-POPS-PIP_2_ vesicles, a value which is compatible with our observations for *K_0.5_*.

Taken together, the results show that PIP_2_ increases the *V_max_* of PKCα and that when its concentration is 5 mol%, the addition of 2 mol% of DOG does not further increase the activity. Moreover, this concentration decreases *K_0.5_* for Ca^2+^ more than 3-fold, almost 5-fold that of DOG and by a half that of POPS. It is also noteworthy that *K_0.5_* values for PIP_2_ amounted to only 0.11 µM in the presence of DOG and 0.39 in its absence, therefore well below the maximum physiological concentration for the inner monolayer of a mammalian plasma membrane. As a consequence, PKCα may be expected to operate near its maximum capacity even in the absence of a cell signal producing diacylglycerol. Nevertheless, we have shown that the presence of DOG may also help, since *K_0.5_* for PIP_2_ notably fell in its presence. On the other hand, since Ca^2+^ has been shown to be essential for the binding of PIP_2_ to the C2 domain of PKCα [4], [54], this enzyme may be triggered simply by an increase in the cytoplasm concentration of this cation. Since it has been shown that the other classical isoenzymes of PKC are similar to PKCα as regards to the affinity of their C2 domains for PIP_2_ [4], the above observations may well be extended to them.

In conclusion, the results obtained in this work are compatible with the sequential mechanism previously proposed (3) and further confirmed in vivo (5). Basically, intracytosolic Ca^2+^ elevations are the trigger to translocate PKCα to the plasma membrane. Once there, two situations can be found: in microdomains enriched only with phosphatidylserine, the docking of the C2 domain is not enough to liberate the catalytic domain for substrate access, and as seen in the 3D structure recently solved [55], the C1B domain might still keep blocking the catalytic domain. Due to this, the presence of 1,2-diacyl-*sn*-glycerol in the lipid vesicles by docking at least the C1A domain enables the enzyme to gain its full activation [56]. A second situation can be found when the microdomains are enriched in phosphatidylserine and PIP_2_ at the plasma membrane. In this case, the C2 domain docks in a different orientation since it has to anchor through two different points, i.e. the CBR (Ca^2+^/PS) and the lysine rich cluster (PIP_2_), this might induce a conformational change that unleash the C1 domain from the blocking conformation and enables the catalytic domain to access the substrate and consequently full activation of the enzyme. Whether the C1 domains can interact with the membrane independently of 1,2-diacyl-*sn*-glycerol is not known but there are previous reports indicating that the C1 domains can interact unspecifically with negatively charged phospholipids through the Arg and Lys residues located in its surface [57].
